# Food Allergy and Mental Health in Children and Adolescents—The Role of Shared Familial Environment

**DOI:** 10.1111/cea.14619

**Published:** 2025-01-15

**Authors:** Hanna Karim, Cecilia Lundholm, Tong Gong, Bronwyn Brew, Michael Silverman, Catarina Almqvist

**Affiliations:** ^1^ Department of Medical Epidemiology and Biostatistics Karolinska Institutet Stockholm Sweden; ^2^ Astrid Lindgren's Children's Hospital Karolinska University Hospital Stockholm Sweden; ^3^ Centre for Big Data Research in Health University of New South Wales Kensington New South Wales Australia; ^4^ School of Medicine and Public Health University of Newcastle Newcastle New South Wales Australia; ^5^ Department of Psychiatry Icahn Medical School at Mount Sinai New York New York USA; ^6^ Pediatric Allergy and Pulmonology Unit, Astrid Lindgren Children's Hospital Karolinska University Hospital Stockholm Sweden

**Keywords:** adolescents, anxiety, children, depression, epidemiology, food allergy, twins

## Abstract

**Background:**

Evidence suggests a link between food allergy and poor mental health, however, this may be explained by shared genetic and environmental factors. We aimed to investigate the association between food allergy of different severity and mental health in children, and the role of familial factors.

**Methods:**

This population‐based, longitudinal cohort study is based on the Child and Adolescent Twin Study in Sweden with questionnaire data reported by parents and/or children. Food allergy ‘ever’ and doctor's diagnosis were reported at age 9–12 years, and ≥ 1 recent dispensation of adrenaline was used as a marker for current severe food allergy. Outcomes were identified using validated questionnaires for anxiety; Screen for Child Anxiety Related Disorders (SCARED); Strength and Difficulties Questionnaire (SDQ) and depression; Short Mood and Feelings Questionnaire (SMFQ), Center for Epidemiological Studies Depression Scale (CES‐D) and Diagnostic and Statistical manual of Mental Disorders (DSM‐IV‐MDE) and reported at 9–12, 15 and 18 years of age. Multivariate linear and logistic modelling was applied to the whole cohort and a co‐twin control approach to remove confounding by familial factors.

**Results:**

In total, 3039 (8.9%) children had a parent‐reported food allergy. Among these, 1292 (43.5%) had non‐severe food allergy without diagnosis, 1490 (49%) had non‐severe food allergy with diagnosis and 257 (8.5%) had severe food allergy. Compared to children with no food allergy, non‐severe food allergy with diagnosis by 9–12 years was associated with parent‐reported anxiety/depression; SCARED (adjOR 2.10, 95% CI 1.48–2.98), SMFQ (adjOR 1.92, 95% CI 1.19–3.10) at 9–12 years and SDQ (adj*β* 0.2, 95% CI 0.0–0.4) at 15 years. All other associations were null including for those with severe food allergy. All positive estimates in the full cohort were attenuated using co‐twin controls.

**Conclusion:**

Evidence associating paediatric food allergy severity and poor mental health was weak, and positive associations observed were likely due to familial confounding.

## Graphical abstract

This population‐based, longitudinal cohort study, found no associations between severe food allergy and poor mental health, but a possible association between non‐severe food allergy and poor mental health at age 9–12 and 15 years. Since all positive estimates were attenuated using co‐twin controls, the possible association seems not to be causal but rather explained by genes and/or familial environmental factors.

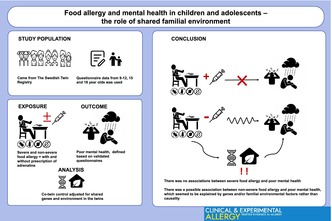

**Summary**

- A possible association between non‐severe food allergy and anxiety/depression in children and adolescents was observed.
- The associations appear to be explained by shared family environment.
- There were no associations between severe food allergy and anxiety/depression.

## 1 Introduction

Food allergy is a common allergic disease in both children and adults with a prevalence of 6%–10% [1, 2, 3, 4]. Food allergy is an immunological reaction that occurs with exposure to a specific food and can be divided into two types; Immunoglobulin E (IgE)‐mediated and non‐IgE‐mediated [5, 6]. The most common form is IgE‐mediated allergy causing several types of allergic reactions: mild, such as pruritus; and severe, such as anaphylaxis [2, 5, 7]. In Western countries, the most common childhood food allergens are milk, eggs, tree nuts, peanuts, fish and shellfish [6]. Many children grow out of their food allergy around school age [1].

The most severe reaction to food allergy—anaphylaxis—often involves several organ systems such as the airways, cardiovascular system, central nervous system, gastrointestinal tract and skin [8]. It may be life‐threating and require emergency treatment with an injection of adrenaline [8]. In Sweden, there are strict criteria for prescription of adrenaline autoinjectors to children, including history of a severe anaphylactic reaction [9]. Except for emergency treatment in case of a severe reaction, there is no other treatment for food allergy. Therefore, affected children need to strictly avoid foods to which they are allergic. However, these behaviours may impact the child's social life and have emotional effects [3].

Several previous studies have shown that food allergy may be associated with anxiety and depression in children [10, 11, 12, 13, 14]. Polloni et al. noted a null or positive association between food allergy and anxiety in their review [11]. A recent study observed an association between food allergy and psychiatric diagnoses [14], although with risk of detection bias due to health‐seeking behaviour. Another study demonstrated a familial aggregation for the comorbidity of atopic diseases and anxiety and depression in children [15], and the association between allergic diseases and poor mental health has been suggested as not causal [16]. Further research on childhood food allergy suggests that anaphylactic reactions to food have a negative impact on mental health [17, 18], but there is a discrepancy on whether prescription of adrenaline is detrimental or helpful to mental health [17, 18, 19]. Childhood food allergy may also have an impact on the whole family, and studies have shown that parents of children with food allergy more often suffer from distress and anxiety [10, 11, 20, 21, 22].

The majority of previous studies on food allergy and mental health are based on small samples, and longitudinal studies have not taken food allergy severity into account [14, 23]. Inconsistency between parental‐ and self‐report on mental health has also been shown. In an Australian cohort study, mothers reported poorer mental health in their food‐allergic adolescents than the adolescents themselves [24]. Furthermore, none of the previous published longitudinal studies have analysed the role of familial factors [14, 23]. One way to investigate this is to use a co‐twin control design that adjusts for all that the twins share, both in their environment (e.g., diet, lifestyle and parental anxiety) and genes [25].

The aim of this study was to investigate if poor mental health in childhood and adolescence is associated with food allergies of different severity and at different ages, and to study if the association can be explained by shared genetics or environment.

## 2 Method

### 2.1 Study Population and Design

Information from the Swedish Twin Registry and the Child and Adolescent Twin Study in Sweden (CATSS) was used. CATSS is a population‐based longitudinal cohort study of Swedish twins born from 1992 onwards [26]. Parents answered a telephone interview (CATSS‐9) when the children were 9 years of age (or 12 years for those born 1992–1995). Children living abroad, with severe disabilities, with parents not fluent in Swedish or who had disabilities making them unable to answer the questionnaire were excluded [27]. Only complete pairs were included. The twins were also contacted for both parental‐ and self‐reported questionnaires at age 15 (CATSS‐15) and self‐report at 18 years (CATSS‐18), irrespective of whether they participated previously.

**FIGURE 1:**
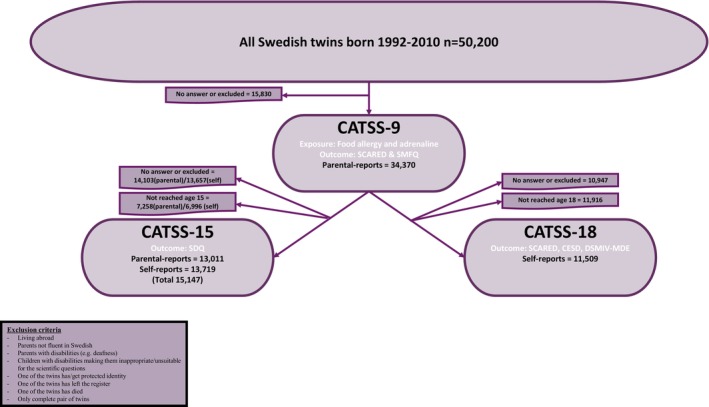
Flowchart of the study population.

The study population for this cohort study included CATSS twins born 1992–2010. In total, 34,370 questionnaires were completed at 9 or 12 [9, 10, 11, 12] years of age (parent‐report), 15,148 at 15 years of age (13,709 self‐report and 13,011 parent‐report) and 13,343 at 18 years of age (self‐report) (Figure 1). Information from CATSS was linked by personal identity number to the Medical Birth Register (MBR), the Swedish Prescribed Drug Register (SPDR) and the National Patient Register (NPR), which are national health registers held by the National Board of Health and Welfare, (see the Supplement). The children in CATSS were also linked to their parents in the Multi‐Generation Register (MGR) held by Statistics Sweden [28].

This study was approved by the Regional Ethical Review Board in Stockholm, Sweden, Dnr 2018/1697‐31/1 (19 September 2018), and the Swedish Ethical Review Authority, Dnr 2021‐04944 (19 October 2021), Dnr 2022‐02568‐02 (20 June 2022). Parents to children in CATSS gave informed consent to link data to the national health registers, and the 18‐years‐old children gave their own informed consent for CATSS‐18. All data was pseudonymised prior to analysis.

### 2.2 Variables

#### 2.2.1 Food Allergy Exposure

Food allergy was identified as either (1) positive response to a question about the child ever having a food allergy other than celiac disease or lactose intolerance by 9–12 years or (2) reported food items in free text as triggers of allergic symptoms ever by 9–12 years. All types of reported food allergens were included, common and uncommon (see Appendix S1). *Non‐severe food allergy* was defined as reported food allergy by 9–12 years of age (CATSS‐9) but no dispensation of adrenaline (ATC‐code C01CA24 from the SPDR). Further, those with *non‐severe food allergy* were divided into: *with* and *without a reported doctor's diagnosis of food allergy* (CATSS‐9). *Severe food allergy* was defined as reported food allergy by 9–12 years old (CATSS‐9) and a concurrent prescription of adrenaline (SPDR), i.e., ≥ 1 dispensation of adrenaline within ±2 years from the CATSS‐9 interview date. All children with severe food allergy were assumed to have a doctor's diagnosis of food allergy since adrenaline is not prescribed otherwise.

#### 2.2.2 Mental Health Outcomes

Different outcomes were defined based on five different validated questionnaires, self‐ and/or parental‐reported, included in the CATSS interviews at 9–12, 15 and 18 years.

Screen for Child Anxiety Related Disorders (SCARED), a screening tool for childhood and adolescent anxiety disorders [29, 30], used at both 9–12 years (*Anxiety‐9*) and 18 years (*Anxiety‐18*) and defined both as continuous (score range 0–82) and categorical measures (validated cut‐off 25) [29].

Short Mood and Feelings Questionnaire (SMFQ), a screening tool for childhood depressive symptoms [31], used at 9–12 years (*Depression‐9*) and defined both as continuous (score range 0–26) and categorical measure (validated cut‐off of 11 for parental‐reported symptoms) [32].

Strength and Difficulties Questionnaire (SDQ), a screening tool for emotional behavioural problems in children [33, 34], used at 15 years (*Anxiety‐/Depression‐15*) and consisting of 5 scales (emotional problems, conduct problems, hyperactivity, peer problems and pro‐social scale). For this study, the emotional scale was used and defined as a continuous measure (score range 0–10) [33].

The shortened Center for Epidemiological Studies Depression scale (CES‐D), a screening tool for depression, used at 18 years (*Depression‐18‐CESD*) and defined as a continuous measure (score range 0–33) [35].

Diagnostic and Statistical Manual of Mental Disorders—Major Depressive Episode (DSM‐IV‐MDE), a diagnostic tool used in clinics when screening for depression [36], used at 18 years (*Depression‐18‐DSM‐IV*) and defined both as continuous (possible score range 0–11) and categorical measures (validated cut‐off for Minor depression = 2–4 and for Major depression ≥ 5) [37].

#### 2.2.3 Covariates

The covariates were selected based on a Directed Acyclic Graph (DAG), (Figure S1) [38], including sex, gestational length, maternal birth country, maternal body mass index (BMI), parity, highest education of the mother when the children were 9–12 years old, and parental hospitalisation – outpatient or unplanned hospital visit for anxiety (ICD10‐code F41) and/or depression (ICD10‐code F32) prior to responding to the CATSS‐9 questionnaire. The covariates were retrieved from the MBR, except maternal education (CATSS) and parental psychiatric diagnoses (NPR).

### 2.3 Statistical Analysis

Linear regression (continuous outcomes) and logistic regression (categorical outcomes) were used to estimate the association between food allergy and poor mental health in the whole cohort. Cluster robust standard errors (sandwich estimators) were used to account for dependency between observations within twin pairs. Estimates were adjusted for all covariates listed above. Results were considered as statistically significant at the 5% level if the 95% confidence interval (CI) for an odds ratio (OR) did not include 1.0, and if the 95% CI for a beta (*β*) level did not include 0.0.

Then, if statistically significant associations were found in the full cohort, co‐twin control analyses were performed using fixed effects linear regression (conditional likelihood) adjusted for sex to estimate the associations with continuous outcomes accounting for measured and unmeasured factors (genetic and environmental) that are shared within twin pairs.

Since adrenaline also can be prescribed in case of allergic reaction to insects, a sensitivity analysis was performed excluding all children with parental‐reported insect allergy (CATSS‐9). A sensitivity analysis was performed excluding all 12‐year‐old children from the 9–12 year old group. Further, a summarising analysis of any food allergy, not taking severity into account, was also done to get an overall assessment.

Spearman correlation was used to quantify the association between parental and self‐reported *Anxiety‐/Depression‐15* at 15 years.

Statistical analyses were conducted using Stata Statistical Software: Release 17, College Station, TX: StataCorp LLC.

## 3 Results

In total, 3039 (8.9%) of 9–12‐year‐old children in CATSS had parent‐reported food allergy. Among these, 1292 (43.5%) had non‐severe food allergy without doctor's diagnosis, 1490 (49.0%) had non‐severe food allergy with doctor's diagnosis and 257 (8.5%) had severe food allergy. Most descriptive characteristics of the children were similar by severity, although mothers of children with severe food allergy were slightly more likely to have lower antenatal BMI and be more highly‐educated, compared to both of the non‐severe food allergy groups and the no food allergy group (Table 1). Prevalence of specific food allergy types and dispensation of adrenaline prescriptions at different ages by food allergy severity are listed in Table S1.

**TABLE 1:** Summary characteristics for the CATSS cohort divided into food allergy severity groups by age 9–12 years.

	No food allergy (*n* = 31,218)		Non‐severe food allergy WITHOUT doctor's diagnosis (*n* = 1292)		Non‐severe food allergy WITH doctor's diagnosis (*n* = 1490)		Severe food allergy (*n* = 257)	
	No.	%	No.	%	No.	%	No.	%
*Child's gender*								
Boy	15,789	50.6	610	47.2	825	55.4	137	53.3
Girl	15,429	49.4	682	52.8	665	44.6	120	46.7
*Gestational length*								
Extreme preterm, ≤ 27 weeks	402	1.3	14	1.1	8	0.5	3	1.2
Very preterm, weeks 28–31	1535	4.9	66	5.1	59	4.0	8	3.1
Moderate preterm, weeks 32–36	10,771	34.5	413	32.0	528	35.4	100	38.9
Term or later, ≥ 37 weeks	17,909	57.4	776	60.1	871	58.5	141	54.9
Missing	601	1.9	23	1.8	24	1.6	5	1.9
*Birth country of the mother*								
Sweden	27,389	87.7	1148	88.9	1348	90.5	228	88.7
Other Nordic country	679	2.2	37	2.9	33	2.2	4	1.6
Europe, North America or Oceania	1062	3.4	30	2.3	30	2.0	5	1.9
Other parts of the world	1475	4.7	55	4.3	59	4.0	15	5.8
Missing	613	2.0	22	1.7	20	1.3	5	1.9
*Parity*								
0	14,168	45.4	665	51.5	736	49.4	141	54.9
1	10,788	34.6	393	30.4	556	37.3	82	31.9
2	4055	13.0	163	12.6	128	8.6	20	7.8
3 or more	1657	5.3	51	3.9	50	3.4	9	3.5
Missing	550	1.8	20	1.5	20	1.3	5	1.9
*Maternal BMI at first antenatal visit*								
Underweight, BMI < 18.5	503	1.6	24	1.9	17	1.1	6	2.3
Normal, BMI 18.5–24.9	16,498	52.8	692	53.6	789	53.0	147	57.2
Overweight, BMI 25.0–29.9	6554	21.0	264	20.4	322	21.6	43	16.7
Obese, BMI > 30.0	2576	8.3	102	7.9	131	8.8	12	4.7
Missing	5087	16.3	210	16.3	231	15.5	49	19.1
*Highest education of the mother when the children were 9–12 years old*								
Primary school	1183	3.8	41	3.2	50	3.4	8	3.1
Upper secondary school	13,513	43.3	532	41.2	566	38.0	91	35.4
Higher education	14,811	47.4	631	48.8	796	53.4	152	59.1
Missing	1711	5.5	88	6.8	78	5.2	6	2.3
*Paternal hospitalisation, outpatient or unplanned hospital visit for depression and/or anxiety*								
	485	1.6	28	2.2	31	2.1	4	1.6
*Maternal hospitalisation, outpatient or unplanned hospital visit for depression and/or anxiety*								
	766	2.5	38	2.9	38	2.6	8	3.1

Abbreviation: BMI, body mass index.

**FIGURE 2:**
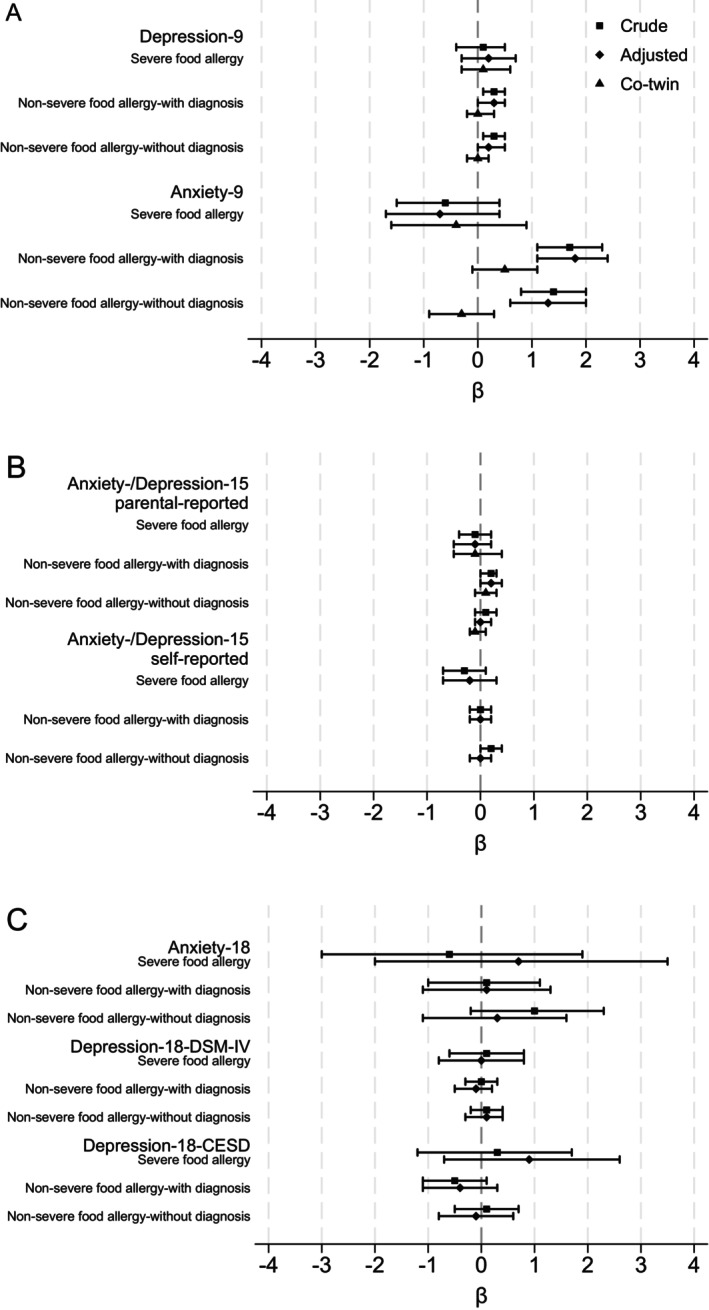
Regression coefficients for crude, adjusted and co‐twin control analyses with 95% confidence intervals by age with no food allergy as reference: (A) 9–12 years, (B) 15 years and (C) 18 years.

When analysing the 9–12 year old outcomes there were no statistically significant associations between *non‐severe food allergy without diagnosis* and poor mental health measured categorically, adjusted odds ratios (adjOR) *Anxiety‐9* (adjOR 1.50, 95% CI 0.97, 2.30) and *Depression‐9* (adjOR 0.99, 95% CI 0.50, 1.96), but there were associations when measured as a continuous measure: *non‐severe food allergy without diagnosis* had adjusted beta (adj*β*) 1.3 (95% CI 0.6, 2.0) higher score on the *Anxiety‐9* and adj*β* 0.2 (95% CI 0.0, 0.5) higher score on *Depression‐9* (Table 2, Figure 2A). There were associations between having *non‐severe food allergy with diagnosis* and both categorical measures of poor mental health: *Anxiety‐9* (adjOR 2.10, 95% CI 1.48, 2.98) and *Depression‐9* (adjOR 1.92, 95% CI 1.19, 3.10) (Table 2, Figure 2A). Similarly, for *Anxiety‐9* and *Depression‐9* as continuous measures (adj*β* 1.8, 95% CI 1.1, 2.4 and adj*β* 0.3, 95% CI 0.0, 0.5). There were no statistically significant associations between *severe food allergy* and any measures of poor mental health (Table 2, Figure 2A).

**TABLE 2:** Association between food allergy by 9–12 years and poor mental health at ages 9–12, 15 and 18 years, and co‐twin control analyses of the outcomes with significant associations.

	Categorical				Continuous					
	Crude		Adjusted		Crude		Adjusted		Co‐twin control	
	OR	95% CI	OR	95% CI	*β*	95% CI	*β*	95% CI	*β*	95% CI
Results 9–12 year olds										
*Anxiety‐9*										
No food allergy	1.00	[1.00, 1.00]	1.00	[1.00, 1.00]	0.0	[0.0, 0.0]	0.0	[0.0, 0.0]	0.0	$\left[0.0,0.0\right]$
Non‐severe food allergy WITHOUT doctor's diagnosis	1.59	[1.09, 2.33]	1.50	[0.97, 2.30]	1.4	[0.8, 2.0]	1.3	[0.6, 2.0]	−0.3	[−0.9, 0.3]
Non‐severe food allergy WITH doctor's diagnosis	1.93	[1.39, 2.68]	2.10	[1.48, 2.98]	1.7	[1.1, 2.3]	1.8	[1.1, 2.4]	0.5	[−0.1, 1.1]
Severe food allergy	0.91	[0.34, 2.46]	0.82	[0.25, 2.65]	−0.6	[−1.5, 0.4]	−0.7	[−1.7, 0.4]	−0.4	[−1.6, 0.9]
*Depression‐9*										
No food allergy	1.00	[1.00, 1.00]	1.00	[1.00, 1.00]	0.0	[0.0, 0.0]	0.0	[0.0, 0.0]	0.0	$$\left[\begin{array}{c} 0.0,0.0 \end{array}\right]$$
Non‐severe food allergy WITHOUT doctor's diagnosis	1.14	[0.63, 2.08]	0.99	[0.50, 1.96]	0.3	[0.1, 0.5]	0.2	[0.0, 0.5]	0.0	[−0.2, 0.2]
Non‐severe food allergy WITH doctor's diagnosis	1.95	[1.28, 2.99]	1.92	[1.19, 3.10]	0.3	[0.1, 0.5]	0.3	[0.0, 0.5]	0.0	[−0.2, 0.3]
Severe food allergy	1.23	[0.39, 3.85]	1.47	[0.47, 4.59]	0.1	[−0.4, 0.5]	0.2	[−0.3, 0.7]	0.1	[−0.3, 0.6]
Results 15 year olds										
*Anxiety‐/Depression‐15 self‐reported*										
No food allergy	NA	NA	NA	NA	0.0	[0.0, 0.0]	0.0	[0.0, 0.0]	NA	NA
Non‐severe food allergy WITHOUT doctor's diagnosis	NA	NA	NA	NA	0.2	[−0.0, 0.4]	0.0	[−0.2, 0.2]	NA	NA
Non‐severe food allergy WITH doctor's diagnosis	NA	NA	NA	NA	−0.0	[−0.2, 0.2]	−0.0	[−0.2, 0.2]	NA	NA
Severe food allergy	NA	NA	NA	NA	−0.3	[−0.7, 0.1]	−0.2	[−0.7, 0.3]	NA	NA
*Anxiety‐/Depression‐15 parental‐reported*										
No food allergy	NA	NA	NA	NA	0.0	[0.0, 0.0]	0.0	[0.0, 0.0]	0.0	$$\left[\begin{array}{c} 0.0,0.0 \end{array}\right]$$
Non‐severe food allergy WITHOUT doctor's diagnosis	NA	NA	NA	NA	0.1	[−0.1, 0.3]	0.0	[−0.1, 0.2]	−0.1	[−0.2, 0.1]
Non‐severe food allergy WITH doctor's diagnosis	NA	NA	NA	NA	0.2	[0.0, 0.3]	0.2	[0.0, 0.4]	0.1	[−0.1, 0.3]
Severe food allergy	NA	NA	NA	NA	−0.1	[−0.4, 0.2]	−0.1	[−0.5, 0.2]	−0.1	[−0.5, 0.4]
Results 18 year olds										
*Depression‐18‐CESD*										
No food allergy	NA	NA	NA	NA	0.0	[0.0, 0.0]	0.0	[0.0, 0.0]	NA	NA
Non‐severe food allergy WITHOUT doctor's diagnosis	NA	NA	NA	NA	0.1	[−0.5, 0.7]	−0.1	[−0.8, 0.6]	NA	NA
Non‐severe food allergy WITH doctor's diagnosis	NA	NA	NA	NA	−0.5	[−1.1, 0.1]	−0.4	[−1.1, 0.3]	NA	NA
Severe food allergy	NA	NA	NA	NA	0.3	[−1.2, 1.7]	0.9	[−0.7, 2.6]	NA	NA
*Depression‐18‐DSM‐IV* *Minor depression criteria*										
No food allergy	1.00	[1.00, 1.00]	1.00	[1.00, 1.00]	0.0	[0.0, 0.0]	0.0	[0.0, 0.0]	NA	NA
Non‐severe food allergy WITHOUT doctor's diagnosis	1.26	[0.86, 1.87]	1.23	[0.79, 1.93]	0.1	[−0.2, 0.4]	0.1	[−0.3, 0.4]	NA	NA
Non‐severe food allergy WITH doctor's diagnosis	0.94	[0.62, 1.44]	0.89	[0.55, 1.43]	−0.0	[−0.3, 0.3]	−0.1	[−0.5, 0.2]	NA	NA
Severe food allergy	0.73	[0.26, 2.02]	0.49	[0.12, 2.04]	0.1	[−0.6, 0.8]	−0.0	[−0.8, 0.8]	NA	NA
*Depression‐18‐DSM‐IV* *Major depression criteria*										
No food allergy	1.00	[1.00, 1.00]	1.00	[1.00, 1.00]	NA	NA	NA	NA	NA	NA
Non‐severe food allergy WITHOUT doctor's diagnosis	1.06	[0.81, 1.38]	1.04	[0.76, 1.40]	NA	NA	NA	NA	NA	NA
Non‐severe food allergy WITH doctor's diagnosis	0.95	[0.73, 1.24]	0.87	[0.63, 1.20]	NA	NA	NA	NA	NA	NA
Severe food allergy	1.05	[0.60, 1.82]	1.04	[0.51, 2.12]	NA	NA	NA	NA	NA	NA
*Anxiety‐18*										
No food allergy	1.00	[1.00, 1.00]	1.00	[1.00, 1.00]	0.0	[0.0, 0.0]	0.0	[0.0, 0.0]	NA	NA
Non‐severe food allergy WITHOUT doctor's diagnosis	1.28	[1.01, 1.62]	1.16	[0.87, 1.55]	1.0	[−0.2, 2.3]	0.3	[−1.1, 1.6]	NA	NA
Non‐severe food allergy WITH doctor's diagnosis	0.98	[0.77, 1.26]	1.02	[0.77, 1.35]	0.1	[−1.0, 1.1]	0.1	[−1.1, 1.3]	NA	NA
Severe food allergy	1.01	[0.60, 1.72]	1.43	[0.77, 2.66]	−0.6	[−3.0, 1.9]	0.7	[−2.0, 3.5]	NA	NA

Abbreviations: CESD, Center for Epidemiological Studies Depression scale; CI, confidence interval; DSM, Diagnostic and Statistical Manual of Mental Disorders; NA, not applicable.

For the 15 year old outcomes, there were no statistically significant associations between food allergy of any severity grade by age 9–12 years and self‐reported poor mental health (Table 2, Figure 2B). Similarly, analysis of parental‐reported mental health did not find any associations between food allergy and *Anxiety‐/Depression‐15* except for a slightly positive association for *non‐severe food allergy with diagnosis* (adj*β* 0.2, 95% CI 0.0, 0.4).

In the 18 year old outcomes there was no statistically significant association between food allergy of any severity grade by age 9–12 years and any measure of poor mental health (Table 2, Figure 2C).

Estimates from the co‐twin control analyses of the outcomes with statistically significant associations in the full cohort (i.e., *Anxiety‐9*, *Depression‐9* and *Anxiety‐/Depression‐15*—parental‐reported) were attenuated and no longer statistically significant, for example in the co‐twin control analysis the group of 9–12 year old children with *non‐severe food allergy with diagnosis* had adj*β* 0.5 (95% CI −0.1, 1.1) higher mean score in *Anxiety‐9* (Table 2).

The sensitivity analysis, excluding children with reported insect allergy, did not show any differences compared to the main results, for example in the group of 9–12‐year‐old children with *severe food allergy* in *Anxiety‐9* adj*β* −0.6 (95% CI −1.7, 0.4) (Table S2). The sensitivity analysis, excluding the 12‐year‐old children from the 9–12 year old group did not show any differences compared to the main results (Table S3).

The result of the analysis of any food allergy, not taking severity into account, showed the same trends as the main analysis; statistically significant associations between food allergy by age 9–12 years and anxiety/depression in the parental reports at 9–12 and 15 years of age, but not for the other outcomes or the co‐twin control, and it can be found in the supplement (Table S4).

Spearman correlation between parental and self‐reported *Anxiety‐/Depression‐15* was *r* = 0.38.

## 4 Discussion

We found a weak association between non‐severe food allergy by 9–12 years and some parent‐reported poor mental health outcomes at age 9–12 and 15 years but none for self‐reported, and no evidence for trends by food allergy severity. All positive associations were attenuated and not statistically significant in the co‐twin control analyses, suggesting that shared genetic or environmental factors likely contributed to the positive findings observed.

The link between food allergy and poor mental health is indeterminate [10, 11, 12, 13, 39, 40]. Polloni et al. reviewed the relationship in both children and adults and concluded that the association might be more of a food allergy‐specific worry, rather than a predisposition towards anxiety [11]. Golding et al. reviewed both quantitative and qualitative studies on poor mental health and quality of life in children and adolescents with food allergy finding similar results [13]. Several longitudinal studies have found conflicting findings; Shanahan et al. studied 1420 adolescents aged 10 to 16 and found an association between parent‐reported food allergy and poor mental health, but not psychiatric diagnoses [23]. Nemet et al., however, observed an association between food allergy and psychiatric diagnoses, based on a population‐based, matched‐cohort study consisting of 603,257 individuals, of whom the majority were children [14]. They had however only register‐based information on food allergy and psychiatric disorders from hospitals and not self‐/parental‐reports, so there is a risk of detection bias based on health‐seeking behaviour. The causality of the association between allergic disease and poor mental has previously been studied and suggested as unlikely [16]. Our study confirms and expands on previous findings addressing possible familial confounding from genes and shared environment [15]. As such, we found that the association between parent‐reported food allergy and poor mental health was weak, and further attenuated when accounting for genes and environment shared within twin pairs in the co‐twin control analysis, indicating that the association is likely not to be causal.

Regarding food allergy severity, a few studies have observed an association between anaphylaxis to food and poor mental health outcomes [17, 18, 19]. Previous studies have shown that both paediatric patients and their parents, and adult patients, can suffer from acute stress after an anaphylactic reaction [41, 42, 43]. The fact that we do not see an association between food allergy and mental health suggests that this stress reaction may not translate into ongoing mental health issues, or possibly that the participants have received previous treatment for this stress. Unfortunately, we do not have this information, but medications for anxiety and depression in children in Sweden are extremely rare. An Italian study on children and adolescents (*n* = 232) aged 11–17 years reported that both previous anaphylactic reactions and adrenaline prescription were associated with poor mental health as measured by SDQ [18]. On the other hand, a cross‐sectional study of British children (*n* = 41) aged 6–16 years and their mothers, found no link between severe food allergy symptoms and poor mental health, but rather, that access to adrenaline autoinjectors can have a positive impact [19]. Similarly, we did not find evidence for trends by food allergy severity grade. This may indicate that the health care system in Sweden and United Kingdom is responding well to food allergy. In Sweden, like the United Kingdom, health care for children is free, including medications such as adrenaline, and allergy‐friendly food is widely available. It is also likely that since food allergy is very common in these countries, children experiencing food allergy are not treated differently to their peers, are well catered for, and thereby not affected in their mental health. Alternatively, since we defined severity based on adrenaline prescription, the lack of association for severity could be explained by the knowledge of treatment availability in case of a severe allergic reaction.

As well as being able to study a number of different parent‐ and self‐reported mental health outcomes at different ages, a novelty of our study was the ability to account for familial (genetic or shared environmental) confounding. The attenuated associations of the co‐twin control analyses compared to the full cohort suggest that there are familial factors that may explain any observed associations between non‐severe food allergy and poor mental health, rather than a causal relationship. This is in line with Brew et al. [15] who found a familial aggregation between food allergy in children and depression and anxiety, and indicates that previous studies [10, 11, 12, 13, 18, 23, 39, 40, 44] may be confounded by genetic and shared environmental factors. Such factors could be shared diet, life style and parental anxiety, especially in younger children. Studies have shown that parents of children with food allergy more commonly suffer from distress and anxiety [10, 20, 21, 22] particularly when the child has severe food allergy [20, 45, 46]. Furthermore, parents of food‐allergic children experience mental health impact of food allergy to a larger degree than the children themselves [13], and parents, especially mothers, can report poor mental health in their offspring even when the children and adolescents do not [13, 24, 47, 48]. Similarly, our study observed an association between food allergy and parental‐reported poor mental health at age 15 but not in child self‐reported mental health.

### 4.1 Strengths and Limitations

To the best of our knowledge, this study is the only longitudinal population‐based twin study on food allergy and mental health in children and adolescents and has several strengths. The study was based on a large cohort of twins who were followed up longitudinally, from childhood to adolescence and late teenage years. Another strength is that the cohort was able to be linked with several national health registers, which provided adequate power to investigate severity grades of food allergy. Further, we could adjust for unmeasured familial confounders through a co‐twin control analysis. Finally, we were able to test and compare a number of both self‐ and parental‐reported outcomes.

There are however some limitations. Firstly, we may have missed those children who, in between the follow‐ups, have had contact with health care because of poor mental health, been treated, and thereby were in good mental health when answering the questionnaire. This would lead to an underestimation of the associations. Further, there may be exposure misclassification, since the ‘no food allergy group’ may not be completely free from food allergies and the ‘severe food allergy group’ may include other allergies causing prescription of adrenaline and adrenaline beyond the Swedish guidelines. However, the sensitivity analysis excluding children with insect allergy did not change the main analysis result and most likely the incorrect prescriptions are not large enough to affect the results. Also, we did not have information on parental postnatal smoking so we were unable to adjust for it in the main analysis. However, we do adjust for parental postnatal smoking by design in the co‐twin control analyses. Further, the confidence intervals for the associations between severe food allergy and categorical outcome variables indicated low power to detect small differences, while the power for the continuous outcomes was better. Moreover, we could not account for mental health before the food allergy onset in the children since we did not have that information. Different questionnaires were used to measure poor mental health changes at the different ages, to suit the age of the child.

### 4.2 Future Studies and Clinical Implication

Our study indicates that the weak association between food allergy and childhood poor mental health may be due to genetic and familial factors. This knowledge may be helpful for parents and clinicians to recognise these factors and to help improve mental health. The results should also be seen in light of the paradigm shift for food allergies, with more readily available oral immunotherapy. Further studies are needed to tease this out further, and to determine the separate roles of access to health care, social stigma around food allergy, genetic factors and familial environment.

## 5 Conclusion

In conclusion, this study found no associations between severe food allergy and poor mental health but a possible association between parent‐reported non‐severe food allergy by 9–12 years and poor mental health at 9–12 and 15 years of age, which appears to be explained by genes and/or familial environmental factors. The lack of a negative effect of severe food allergy on mental health may be the result of a well‐functioning health care system or a psychological effect of having access to an emergency treatment (adrenaline autoinjector) in case of a severe reaction. These hypotheses require further research.

## Author Contributions

Study concept and design: H.K., C.L., B.B., M.S., C.A. Data acquisition and funding: C.A., B.B. and M.S. Data analysis: H.K., C.L., C.A. Data interpretation: H.K., C.L., T.G., B.B., M.S., C.A. Initial draft of the manuscript: H.K., C.A. All authors critically reviewed and revised the manuscript and approved the final version. The corresponding author attest that all listed authors meet authorship criteria and that no others meeting the criteria have been omitted.

## Conflicts of Interest

The authors declare no conflicts of interest.

## Supporting information


Appendix S1

## Data Availability

Original data are held by the Swedish Twin Registry and the National Board of Health and Welfare. Due to the Swedish data storage laws the data cannot be made publicly available. However, any researcher can access the data by obtaining an ethical approval from the Swedish Ethical Review Authority and request the original data from the register holders. Pseudonymised data may be provided by the corresponding author upon reasonable requests and if an appropriate data sharing agreement with Karolinska Institutet is established.
