# Supplementary material for: Food Allergy and Mental Health in Children and Adolescents—The Role of Shared Familial Environment
Source: Clin Exp Allergy. 2025 Jan 15;55(2):175–86. doi: 10.1111/cea.14619 (PMC11791381; doi:10.1111/cea.14619)
Supplement: Supplementary file 1 — Appendix S1 [file CEA-55-175-s001.docx]

**Supplement**

**Food allergy and mental health in children and adolescents - the role of shared familial environment**

Hanna Karim, Cecilia Lundholm, Tong Gong, Bronwyn Brew, Michael Silverman, Catarina Almqvist

**Method**

- 1. **Food allergy exposure**
  2. **Study population and design**

**Results**

**Table S1.** Descriptive of the allergies in the CATSS cohort age 9-12 years, divided into food allergy severity groups

**Table S2.** Sensitivity analysis with parental reported insect allergy cases excluded

**Table S3.** Sensitivity analysis with 12 year old children excluded from the 9-12 year old group.

**Table S4.** Analysis of any food allergy

**Figure S1.** Directed Acyclic Graph (DAG) with the selected covariates

This supplemental material has been provided by the authors to give readers additional information about their work

**Method**

**1.1 Food allergy exposure**

Food allergy was identified as either 1) a positive response to a question about the child ever having a food allergy other than celiac disease or lactose intolerance by 9-12 years or 2) reported food items in free text as triggers of allergic symptoms ever by 9-12 years. All types of reported food allergens were included, common and uncommon. Information about allergen specific food allergy diagnosis and diagnosis of anaphylactic reactions was retrieved from the National Patient Register (NPR). Information about dispensed adrenaline was retrieved from the Swedish Prescribed Drug Register (SPDR).

**Covariates**

The covariates were selected based on a Directed Acyclic Graph (DAG), (**Figure S1)** (1), including: sex, gestational length, maternal birth country, maternal body mass index (BMI), parity, highest education of the mother when the children were 9-12 years old, and parental hospitalisation- outpatient or unplanned hospital visit for anxiety (ICD10-code F41) and/or depression (ICD10-code F32) prior to responding to the CATSS-9 questionnaire. The covariates were retrieved from the MBR, except maternal education (CATSS) and parental psychiatric diagnoses (NPR).

**1.3 Study population and design**

The Medical Birth Register contains data from 97% of all deliveries in Sweden since 1973 and includes prenatal, delivery and neonatal care data (2). SPDR contains information of all prescribed drugs dispensed at pharmacies since 2005, coded according to the Anatomical Therapeutic Chemical (ATC) classification system (3). NPR includes all in-patient care (since 1987) and outpatient care (since 2001), including diagnoses coded with the International Classification of Disease version 10 (ICD-10) (4).

**Results**

In the group of children with *non-severe food allergy* *without diagnosis*, the most common questionnaire reported allergen was tree nut followed by milk and in the group with *non-severe food allergy with diagnosis*, the most common allergen was milk protein, followed by egg (**Table S1**). In children with *severe food allergy* the most common reported allergens were peanuts and tree nuts.

Among children with *no food allergy* by 9-12 years, 78 (0.2%) had dispensed prescription of adrenaline. Among the children with *severe food allergy* at 9-12 years, 98 (38.1%) had dispensed prescriptions of adrenaline when 15 years old, and 73 (28.4%f) at 18 years of age. Comorbidity with insect allergy was highest among children in the *severe food allergy* group (2.3%)*.*

None of the sensitivity analysis (**Table S2** and **Table S3**) changed the main results. Excluding children with parent-reported insect allergy (**Table S2**) and 12 year olds from the 9-12 year old children (**Table S3**) respectively still showed statistically significant results for the associations between parental reports of food allergy and the outcomes.

The result of the analysis of any food allergy, not taking severity into account (**Table S4**), showed the same trends as the main analysis; statistically significant associations between food allergy by age 9-12 years and anxiety/depression in the parental reports at 9-12 and 15 years of age, but not for the other outcomes or the co-twin control.

**Table S1. Descriptive of the allergies in the CATSS cohort age 9-12 years, divided into food allergy severity groups**

|  | No food allergy | | Non-severe food allergy WITHOUT doctor’s diagnosis | | Non-severe food allergy WITH doctor’s diagnosis | | Severe food allergy | |
| --- | --- | --- | --- | --- | --- | --- | --- | --- |
|  | No. | % | No. | % | No. | % | No. | % |
| All children | 31218 | 100.0 | 1292 | 100.0 | 1490 | 100.0 | 257 | 100.0 |
| DIFFERENT TYPE OF FOOD ALLERGIES REPORTED IN QUESTIONNAIRE | | | | | | | | |
| Yes | 0 | 0.0 | 42 | 3.3 | 371 | 24.9 | 76 | 29.6 |
|  | 0 | 0.0 | 17 | 1.3 | 7 | 0.5 | 1 | 0.4 |
| Fish allergy reported in questionnaire | | | | | | | | |
| Yes | 0 | 0.0 | 6 | 0.5 | 11 | 0.7 | 2 | 0.8 |
|  | 0 | 0.0 | 19 | 1.5 | 7 | 0.5 | 1 | 0.4 |
| Shellfish allergy reported in questionnaire | | | | | | | | |
| Yes | 0 | 0.0 | 39 | 3.0 | 35 | 2.3 | 13 | 5.1 |
|  | 0 | 0.0 | 19 | 1.5 | 7 | 0.5 | 1 | 0.4 |
| Peanut allergy reported in questionnaire | | | | | | | | |
| Yes | 0 | 0.0 | 65 | 5.0 | 210 | 14.1 | 163 | 63.4 |
|  | 0 | 0.0 | 19 | 1.5 | 7 | 0.5 | 1 | 0.4 |
| Tree nut allergy reported in questionnaire | | | | | | | | |
| Yes | 0 | 0.0 | 159 | 12.3 | 333 | 22.3 | 147 | 57.2 |
|  | 0 | 0.0 | 17 | 1.3 | 7 | 0.5 | 0 | 0.0 |
| Milk protein allergy reported in questionnaire | | | | | | | | |
| Yes | 0 | 0.0 | 119 | 9.2 | 583 | 39.1 | 56 | 21.8 |
|  | 0 | 0.0 | 3 | 0.2 | 0 | 0.0 | 1 | 0.4 |
| Wheat allergy reported in questionnaire | | | | | | | | |
| Yes | 0 | 0.0 | 1 | 0.1 | 22 | 1.5 | 1 | 0.4 |
|  | 0 | 0.0 | 19 | 1.5 | 7 | 0.5 | 1 | 0.4 |
| Soy allergy reported in questionnaire | | | | | | | | |
| Yes | 0 | 0.0 | 7 | 0.5 | 86 | 5.8 | 49 | 19.1 |
|  | 0 | 0.0 | 19 | 1.5 | 7 | 0.5 | 1 | 0.4 |
| DIFFERENT TYPE OF FOOD ALLERGIES REGISTRATED IN NPR | | | | | | | | |
| Diagnosis of egg allergy in NPR | | | | | | | | |
| Yes | 15 | 0.0 | 7 | 0.5 | 65 | 4.4 | 29 | 11.3 |
| Diagnosis of fish or shellfish allergy in NPR | | | | | | | | |
| Yes | 4 | 0.0 | 0 | 0.0 | 10 | 0.7 | 6 | 2.3 |
| Diagnosis of peanut or tree nut allergy in NPR | | | | | | | | |
| Yes | 11 | 0.0 | 0 | 0.0 | 62 | 4.2 | 79 | 30.7 |
| Diagnosis of milk allergy in NPR | | | | | | | | |
| Yes | 74 | 0.2 | 11 | 0.9 | 76 | 5.1 | 17 | 6.6 |
| Diagnosis of other food allergy in NPR | | | | | | | | |
| Yes | 18 | 0.1 | 2 | 0.2 | 32 | 2.1 | 19 | 7.4 |
| DETAILED INFORMATION ABOUT THE FOOD ALLERGY FROM THE QUESTIONNAIRE | | | | | | | | |
| Still have food allergy when 9-12 years old | | | | | | | | |
| Yes | 0 | 0.0 | 757 | 58.6 | 896 | 60.1 | 229 | 89.1 |
|  | 0 | 0.0 | 161 | 12.5 | 93 | 6.2 | 13 | 5.1 |
| INFORMATION ON ADRENALIN DISPENSATION AT DIFFERENT AGES IN SPDR | | | | | | | | |
| Dispensation of adrenaline in SPDR when 9-12 years old | | | | | | | | |
| Yes | 78 | 0.2 | 0 | 0.0 | 0 | 0.0 | 257 | 100.0 |
| Dispensation of adrenaline in SPDR when 15 years old | | | | | | | | |
| Yes | 66 | 0.2 | 3 | 0.2 | 25 | 1.7 | 98 | 38.1 |
| Dispensation of adrenaline in SPDR when 18 years old | | | | | | | | |
| Yes | 59 | 0.2 | 9 | 0.7 | 35 | 2.3 | 73 | 28.4 |
| INFORMATION ON ANAPHYLAXIS FROM THE NPR | | | | | | | | |
| Diagnosis of anaphylaxis in NPR | | | | | | | | |
| Yes | 3 | 0.0 | 0 | 0.0 | 1 | 0.1 | 12 | 4.7 |
| Diagnosis of anaphylaxis in the past in NPR | | | | | | | | |
| Yes | 3 | 0.0 | 0 | 0.0 | 2 | 0.1 | 14 | 5.4 |
| INFORMATION ABOUR INSECT ALLERGY FROM THE QUESTIONNAIRE | | | | | | | | |
| Insect allergy reported in questionnaire | | | | | | | | |
| Yes | 336 | 1.1 | 24 | 1.9 | 23 | 1.5 | 6 | 2.3 |

Abbreviations: NPR, National Patient Register; SPDR, Swedish Prescribed Drug Register

**Table S2. Sensitivity analysis with parental reported insect allergy cases excluded**

| SENSITIVITY ANALYSIS -INSECT ALLERGY | | |
| --- | --- | --- |
|  | **CONTINUOUS** | |
| Anxiety-9 | **β** | **95% CI** |
| No food allergy | 0.0 |  |
| Non-severe food allergy WITHOUT doctor´s diagnosis | 1.4 | [0.7,2.1] |
| Non-severe food allergy WITH doctor´s diagnosis | 1.8 | [1.1,2.5] |
| Severe food allergy | -0.6 | [-1.7,0.4] |
|  | | |
| Depression-9 | **Β** | **95% CI** |
| No food allergy | 0.0 |  |
| Non-severe food allergy WITHOUT doctor´s diagnosis | 0.3 | [0.0,0.5] |
| Non-severe food allergy WITH doctor´s diagnosis | 0.3 | [0.0,0.5] |
| Severe food allergy | 0.2 | [-0.3,0.7] |
|  | | |
| Anxiety-/Depression-15 self-reported | **β** | **95% CI** |
| No food allergy | 0.0 |  |
| Non-severe food allergy WITHOUT doctor´s diagnosis | 0.0 | [-0.2,0.2] |
| Non-severe food allergy WITH doctor´s diagnosis | -0.0 | [-0.2,0.2] |
| Severe food allergy | -0.2 | [-0.7,0.3] |
|  | | |
| Anxiety-/Depression-15 parental-reported | **β** | **95% CI** |
| No food allergy | 0.0 |  |
| Non-severe food allergy WITHOUT doctor’s diagnosis | 0.0 | [-0.1,0.2] |
| Non-severe food allergy WITH doctor’s diagnosis | 0.2 | [0.0,0.4] |
| Severe food allergy | -0.1 | [-0.5,0.3] |
|  | | |
| Depression-18-CESD | **β** | **95% CI** |
| No food allergy | 0.0 |  |
| Non-severe food allergy WITHOUT doctor’s diagnosis | -0.0 | [-0.7,0.7] |
| Non-severe food allergy WITH doctor’s diagnosis | -0.5 | [-1.2,0.2] |
| Severe food allergy | 1.1 | [-0.6,2.8] |
|  | | |
| Depression-18-DSM-IV | **β** | **95% CI** |
| No food allergy | 0.0 |  |
| Non-severe food allergy WITHOUT doctor’s diagnosis | 0.1 | [-0.3,0.4] |
| Non-severe food allergy WITH doctor’s diagnosis | -0.1 | [-0.5,0.2] |
| Severe food allergy | 0.0 | [-0.8,0.8] |
|  | | |
| Anxiety-18 | **β** | **95% CI** |
| No food allergy | 0.0 |  |
| Non-severe food allergy WITHOUT doctor’s diagnosis | 0.3 | [-1.0,1.7] |
| Non-severe food allergy WITH doctor’s diagnosis | 0.1 | [-1.1,1.3] |
| Severe food allergy | 0.8 | [-2.0,3.5] |

Abbreviations: CI, Confidence intervals

**Table S3. Sensitivity analysis with 12 year old children excluded from the 9-12 year old group.**

| SENSITIVITY ANALYSIS – 12 YEAR OLDS | | |
| --- | --- | --- |
|  | **CONTINUOUS** | |
| Anxiety-9 | **β** | **95% CI** |
| No food allergy | 0.0 |  |
| Non-severe food allergy WITHOUT doctor´s diagnosis | 1.3 | [0.6,2.0] |
| Non-severe food allergy WITH doctor´s diagnosis | 1.8 | [1.1,2.4] |
| Severe food allergy | -0.7 | [-1.7,0.3] |
|  | | |
| Depression-9 | **β** | **95% CI** |
| No food allergy | 0.0 |  |
| Non-severe food allergy WITHOUT doctor´s diagnosis | 0.2 | [0.0,0.5] |
| Non-severe food allergy WITH doctor´s diagnosis | 0.3 | [0.0,0.5] |
| Severe food allergy | 0.2 | [-0.3,0.7] |
|  | | |
| Anxiety-/Depression-15 self-reported | **β** | **95% CI** |
| No food allergy | 0.0 |  |
| Non-severe food allergy WITHOUT doctor´s diagnosis | 0.1 | [-0.1,0.4] |
| Non-severe food allergy WITH doctor´s diagnosis | 0.0 | [-0.2,0.2] |
| Severe food allergy | -0.2 | [-0.8,0.3] |
|  | | |
| Anxiety-/Depression-15 parental-reported | **β** | **95% CI** |
| No food allergy | 0.0 |  |
| Non-severe food allergy WITHOUT doctor’s diagnosis | 0.1 | [-0.1,0.3] |
| Non-severe food allergy WITH doctor’s diagnosis | 0.2 | [0.0,0.4] |
| Severe food allergy | -0.2 | [-0.6,0.2] |
|  | | |
| Depression-18-CESD | **β** | **95% CI** |
| No food allergy | 0.0 |  |
| Non-severe food allergy WITHOUT doctor’s diagnosis | 0.2 | [-0.7,1.0] |
| Non-severe food allergy WITH doctor’s diagnosis | -0.3 | [-1.1,0.5] |
| Severe food allergy | 1.4 | [-0.7,3.6] |
|  | | |
| Depression-18-DSM-IV | **β** | **95% CI** |
| No food allergy | 0.0 |  |
| Non-severe food allergy WITHOUT doctor’s diagnosis | -0.1 | [-0.6,0.3] |
| Non-severe food allergy WITH doctor’s diagnosis | -0.0 | [-0.5,0.4] |
| Severe food allergy | -0.1 | [-1.1,0.9] |
|  | | |
| Anxiety-18 | **β** | **95% CI** |
| No food allergy | 0.0 |  |
| Non-severe food allergy WITHOUT doctor’s diagnosis | -0.2 | [-1.8,1.5] |
| Non-severe food allergy WITH doctor’s diagnosis | 0.6 | [-0.9,2.0] |
| Severe food allergy | 2.1 | [-1.1,5.4] |

Abbreviations: CI, Confidence intervals

**Table S4. Analysis of any food allergy**

|  | **CATEGORICAL** | | | | | **CONTINUOUS** | | | | | | |
| --- | --- | --- | --- | --- | --- | --- | --- | --- | --- | --- | --- | --- |
|  | **CRUDE** | | **ADJUSTED** | | | **CRUDE** | | **ADJUSTED** | | | **Co-TWIN control** | |
|  | **OR** | **95% CI** | **OR** | **95% CI** | **P-value** | **β** | **95% CI** | **β** | **95% CI** | **P-value** | **β** | **95% CI** |
| **Anxiety-9** | 1.70 | [1.32,2.20] | 1.73 | [1.31,2.29] | <0.001 | 1.4 | [1.0,1.8] | 1.4 | [0.9,1.8] | <0.001 | 0.0 | [-0.4,0.5] |
| **Depression-9** | 1.56 | [1.11,2.21] | 1.5 | [1.02,2.22] | 0.041 | 0.3 | [0.1,0.4] | 0.2 | [0.1,0.4] | 0.003 | 0.0 | [-0.1,0.2] |
| **Anxiety-/Depression-15**  **self-reported** | NA | NA | NA | NA | NA | 0.0 | [-0.1,0.2] | -0.0 | [-0.2,0.1] | 0.784 | NA | NA |
| **Anxiety-/Depression-15**  **parental-reported** | NA | NA | NA | NA | NA | 0.1 | [0.0,0.2] | 0.1 | [-0.0,0.2] | 0.100 | -0.0 | [-0.1,0.1] |
| **Depression-18-CESD** | NA | NA | NA | NA | NA | -0.2 | [-0.6,0.3] | -0.1 | [-0.6,0.3] | 0.566 | NA | NA |
| **Depression-18-DSM-IV**  **Minor depression criteria** | 1.06 | [0.80,1.40] | 0.99 | [0.72,1.37] | 0.959 | 0.0 | [-0.2,0.3] | -0.0 | [-0.3,0.2] | 0.822 | NA | NA |
| **Depression-18-DSM-IV**  **Major depression criteria** | 1.01 | [0.84,1.21] | 0.96 | [0.77,1.19] | 0.687 | NA | NA | NA | NA | NA | NA | NA |
| **Anxiety-18** | 1.11 | [0.94,1.31] | 1.12 | [0.92,1.36] | 0.271 | 0.4 | [-0.4,1.2] | 0.3 | [-0.6,1.1] | 0.565 | NA | NA |

Abbreviations: CI, Confidence intervals

**Figure S1. Directed Acyclic Graph (DAG) with the selected covariates**


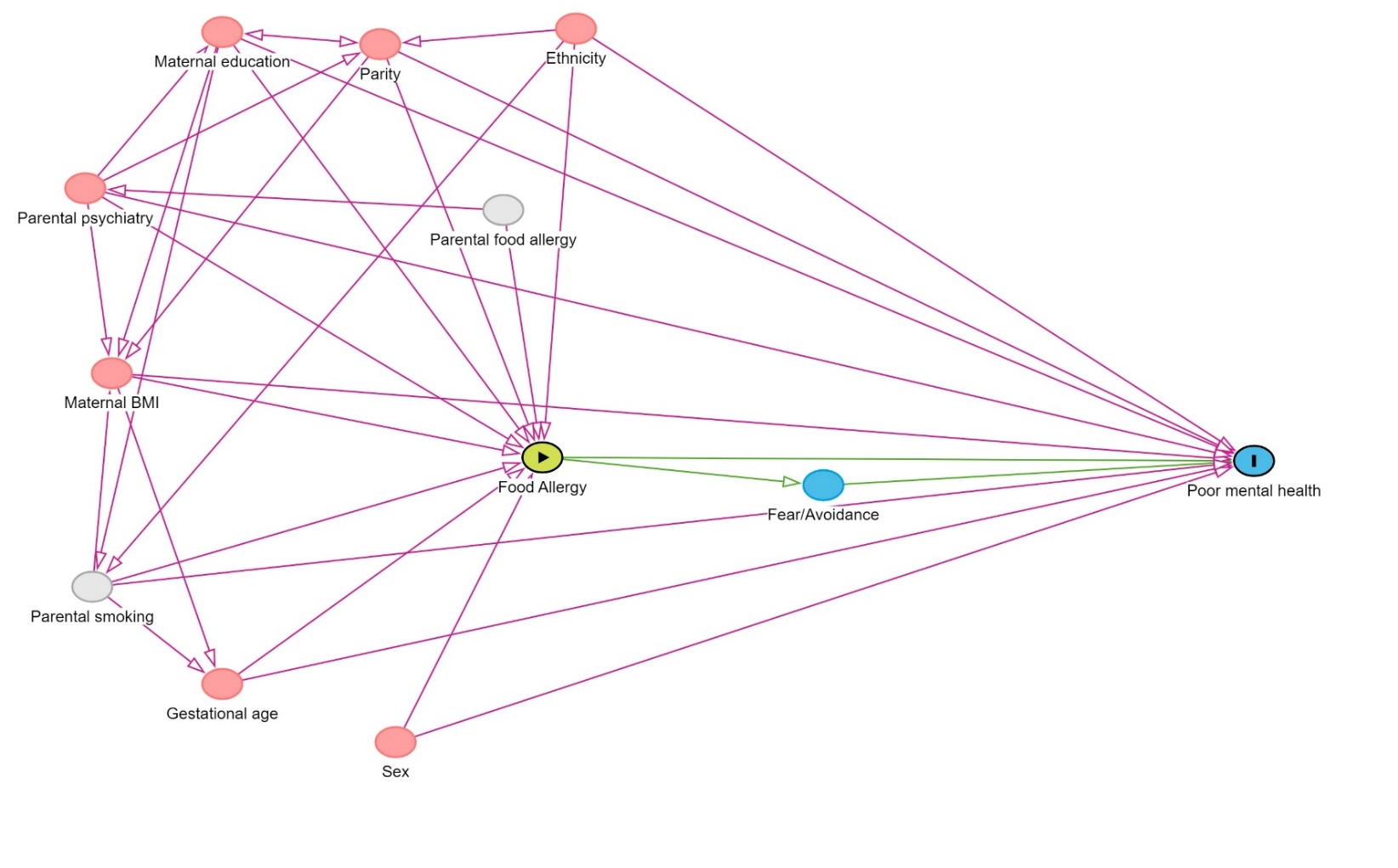


**References**

1. Greenland S, Pearl J, Robins JM. Causal diagrams for epidemiologic research. Epidemiology. 1999;10(1):37-48.

2. Cnattingius S, Källén K, Sandström A, Rydberg H, Månsson H, Stephansson O, et al. The Swedish medical birth register during five decades: documentation of the content and quality of the register. Eur J Epidemiol. 2023;38(1):109-20.

3. Wettermark B, Hammar N, Fored CM, Leimanis A, Otterblad Olausson P, Bergman U, et al. The new Swedish Prescribed Drug Register--opportunities for pharmacoepidemiological research and experience from the first six months. Pharmacoepidemiol Drug Saf. 2007;16(7):726-35.

4. Ludvigsson JF, Andersson E, Ekbom A, Feychting M, Kim JL, Reuterwall C, et al. External review and validation of the Swedish national inpatient register. BMC Public Health. 2011;11:450.
